# Commentary: Coordinating Thalamocortical Connections and Interneuron Migration in the Mammalian Cortex: Role of the *Intermediates*

**DOI:** 10.3389/fncel.2016.00082

**Published:** 2016-03-31

**Authors:** Alejandro López Tobón, Bhavin Shah

**Affiliations:** ^1^Institut für Molekulare Zellbiologie, Westfälische Wilhelms-UniversitätMünster, Germany; ^2^Department of Experimental Oncology, European Institute of Oncology (IEO), Institute of Molecular Oncology Foundation and European Institute of Oncology Campus (IFOM-IEO Campus)Milan, Italy

**Keywords:** neuronal migration, axon development, interneuron, CXCL12, CXCR4, thalamocortical axons, intermediate progenitors

During embryogenesis, thalamocortical axons (TCAs) organize themselves in defined bundles that follow a unique pathway crossing several anatomical boundaries, which makes them easy to track during different stages of neocortical development. These features render TCAs notoriety as a model to study the extrinsic cues that regulate axon guidance and elongation in vertebrate systems. Amid neocortical development, corticothalamic axons (CTAs), project to the subpallium to target the thalamic and subcortical areas, whereas the ascending TCAs project through the ventral telencephalon toward the pallium and spread to reach diverse regions of the neocortex (Garel and Lopez-Bendito, 2014).

TCAs advance through the subpallial region, moving slowly at the pallial-subpallial boundary (PSPB) and meet the corticofugal axons approximately around E14. At around E16, TCAs transit via the intermediate zone (IZ) and ascend to the cortical plate in order to make appropriate networks by creating synaptic contacts to the cortical neurons (Maroof and Anderson, 2006). TCAs trajectory through the subpallium is influenced by locally secreted Netrin-1 (Braisted et al., 2000), followed by progression into the pallium as a response to the timely release of neuregulin, which concomitantly promotes the tangential migration of interneurons (Lopez-Bendito et al., 2006).

Interneurons are generated from several progenitor pools at defined proliferative foci in the ganglionic eminences of the subpallium (Flames et al., 2007). Newly formed INs become bipolar and initiate migration toward the cortex following a characteristic tangential migration. The crosstalk between local repulsion and distal chemo-attraction delineates two clear migratory corridors that enable INs to populate the neocortex, one flowing through the marginal zone (MZ) and the second through the SVZ/IZ (Peyre et al., 2015).

Chemokines containing the CXC motif (conserved cysteine residues separated by an amino acid) play a central role in axonal progression by activating their receptors at axon growth cones, thus triggering a towing mechanism that promotes their elongation and ensures target meeting (Gilmour et al., 2004; Lieberam et al., 2005). In the mammalian cortex, stromal cell-derived factor-1 (SDF-1), also known as CXCL12, along with its receptors CXCR4 and CXCR7, are essential for progress and pathfinding of motor axons (Lieberam et al., 2005). Studies using cultured neurons showed that the CXCL12/CXCR4 pathway leads to an enhanced growth cone dynamics and axon elongation by the activation of a non-conventional pathway that regulates the actin cytoskeleton (Arakawa et al., 2003; Pujol et al., 2005). In addition, *in vivo* analysis of mice with various genetic backgrounds has demonstrated that CXCL12 is indispensable for the migration of neurons that populate the cerebral cortex (Stumm et al., 2003) and multiple other areas in the CNS (Stumm and Hollt, 2007). However, until recently there was no evidence of a specific cell population linking these processes.

In a work published in the Journal of Neuroscience, Abe et al. addressed this question by means of an elegant combination of mouse genetics and fluorescent labeling, thus uncovering a mechanism that links neuronal migration with TCAs progression, through the reciprocal expression of the chemokine CXCL12 in intermediate progenitors cells (IPCs) and its receptor CXCR4 on TCAs (Abe et al., 2015). The work of Abe and colleagues analyzed mouse neocortical development after the conditional ablation and overexpression of Cxcl12 in IPCs. This was achieved by inducing Cre-mediated excision of Cxcl12 encoding exon2 under the control of the Tbr2 promoter in mice. The cortical defects displayed by these mice clearly pinpointed the IPCs as main source of CXCL12. Furthermore, by using a *Cxcl12*^−∕−^ mutant with a Cxcr4-GFP background that specifically traced ascending axons, they found an attenuation of TCA outgrowth, which further confirmed the role of IPCs-derived CXCL12 as a driver of TCAs elongation from the sub-striatal areas. Interestingly, despite the well-known role of CXCL12 in axon pathfinding (Lieberam et al., 2005), the TCAs from *Cxcl12*^−∕−^ mouse brains that reached the IZ at E16.5 showed no signs of misrouting. This discrepancy suggests that the pathfinding effect of CXCL12 may be primed by other unidentified cues regulating TCA trajectory.

In addition to ablation, gain of function experiments underscored the accelerated growth of intra-cortical TCAs via CXCL12/CXCR4. This was achieved by the generation of double transgenic mice that express CXCR4-GFP and CXCL12-RFP under the control of the Cxcl12 promoter, resulting in an endogenous overexpression of the chemokine. These mice showed an enhanced TCA elongation rate as well as an interesting heterotopic TCA branching toward the closest sources of CXCL12 at the meninges. Moreover, the IPC-specific depletion of CXCL12 also led to defects in the interneuron trajectories in the cerebral cortex toward the IZ (absent in the Marginal zone, MZ). These findings clearly stated that the IPC-mediated expression of CXCL12 drives not only proper TCA outgrowth into the neocortex, but also orchestrates a migratory route for the interneurons (Figure 1).

**Figure 1:**
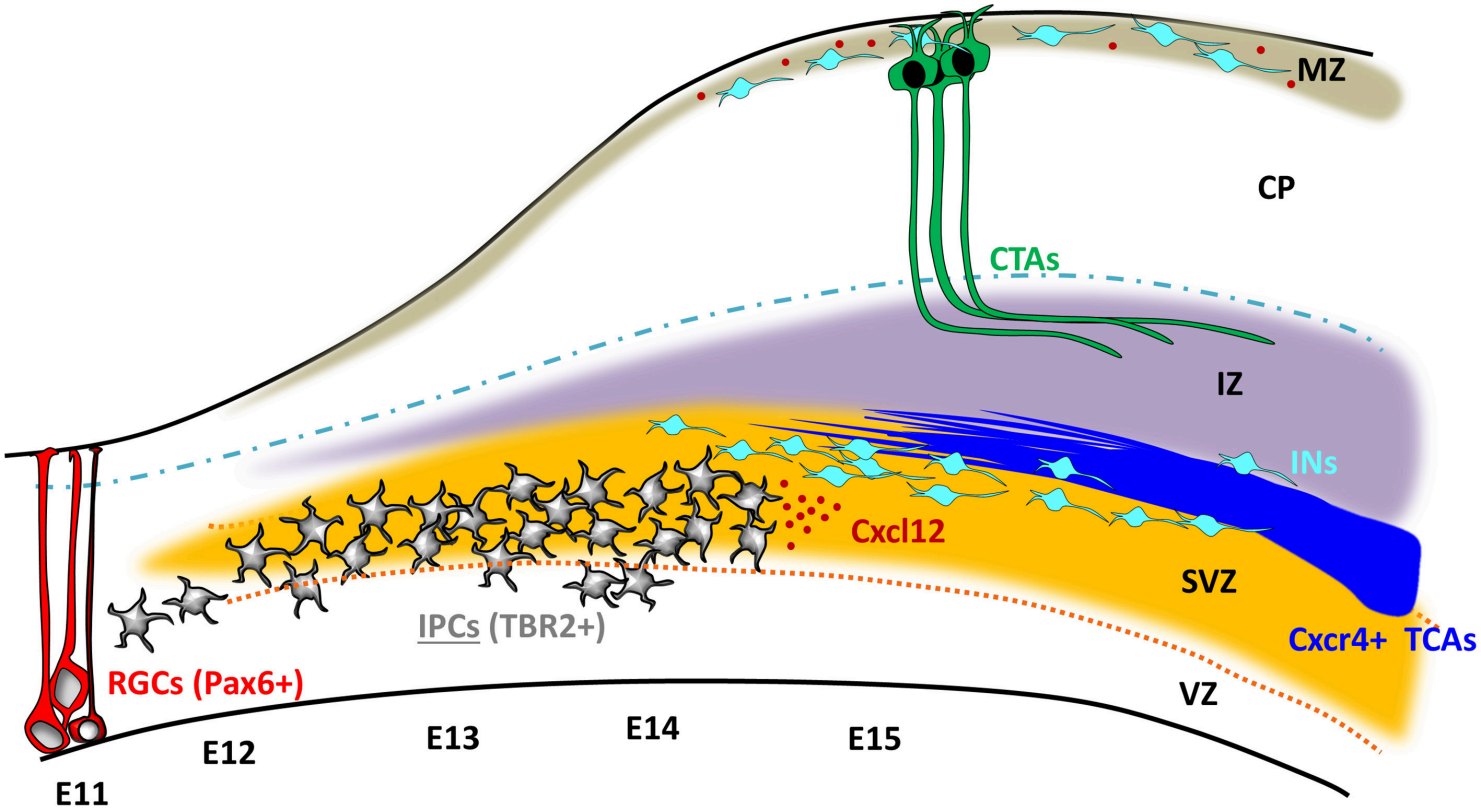
**Intermediate progenitors influence TCA progression and interneuron migration through CXCL12 expression**. At embryonic day 11 (E11), the Pax6^+^ radial glial cells (RGCs; Red) undergo asymmetric divisions that generate a renewed pool of RGCs and Tbr2^+^ committed progenitors (gray) that populate the sub-ventricular zone (SVZ; yellow). Between E11 and E13, the intermediate progenitor cells (IPCs) start to populate the SVZ. During the same period, TCAs (blue) and Interneurons (INs, light blue) expressing CXCR4 exit subcortical areas following a trajectory into the internal capsule. TCAs and INs further advance following the influence of IPC-released CXCL12 gradient (red dots) and meet TCAs approximately at E13.5. From E16 onwards, TCAs spread out of the intermediate zone (IZ; purple) to connect to the principal neurons (green) in the cortical plate (CP).

The picture of INs migration linked to TCA progression remains however incomplete, as the reported migratory defect results from the analysis of the depletion of CXCL12 from IPCs at a single developmental stage. Time-lapse experiments could define the precise dynamics behind the IPC-mediated regulation of INs migration. In addition, the reciprocal effect of TCAs-CTAs in the *Cxcl12* mutants remained unexplored. It is known that the progression of CTAs and TCAs is mutually dependent during cortical development (Hevner et al., 2002; Deck et al., 2013). Whether CTAs show abnormal trajectories or impaired growth in conjunction with the attenuation of intracortical TCA progression could be informative for dissecting the precise role of Cxcl12 in outgrowth and/or pathfinding. Likewise analysis of perinatal stages of neocortical development will be informative to understand the impact of the absence of CXCL12 in the cortical layering after birth, as well as to resolve whether TCA growth is simply delayed or compromised in *Cxcl12* mutants.

The study by Abe et al., suggests the existence of an axis between axon development, neuronal migration and chemotaxis that is centralized in IPCs, opening a new venture in understanding how different aspects of brain development are intermingled. Their findings highlight the role of intermediate progenitors as a source of chemokines stimulating TCA outgrowth and interneuron migration in a coordinated fashion. Whether chemokines influence the precise localization of interneurons at specific cortical layers, as well as the TCA–CTA crosstalk and pathfinding, remains a matter of future investigation.

## Author contributions

AL: Original concept, writing and discussion. BS: Writing, figure design, and discussion.

### Conflict of interest statement

The authors declare that the research was conducted in the absence of any commercial or financial relationships that could be construed as a potential conflict of interest.
